# Mixed Bat‐Hummingbird Pollination Assures Reproductive Success in a Highly Variable Upper Montane Species

**DOI:** 10.1002/ece3.72391

**Published:** 2025-10-23

**Authors:** Isis Paglia, Gabriel Coimbra, Leandro Freitas

**Affiliations:** ^1^ Jardim Botânico Do Rio de Janeiro Rio de Janeiro Brazil

**Keywords:** chiropterophily, mixed pollination systems, ornithophily, pollen tube growth, seed set

## Abstract

*Callianthe sellowiana* is a high‐altitude species endemic to the Atlantic Forest that exhibits floral traits overlapping between ornithophily (hummingbird pollination) and chiropterophily (bat pollination), with highly variable flower color and shape and a yet unstudied reproductive system. We conducted observations and experiments in the upper montane Brazilian Atlantic Forest (2000 m a.s.l.), which revealed that both bat and hummingbird pollinators contribute similarly to fruit and seed set, with no significant difference between diurnal and nocturnal exclusion treatments. However, combined pollination yielded higher reproductive success, indicating functional complementarity and equal pollination effectiveness. Floral traits match both pollinators, with wider corollas at night aiding bat access and enhancing acoustic signals and narrower corollas during the day facilitating hummingbird pollination. Spectral analyses revealed low UV reflectance with a peak in red wavelengths, and for most phenotypes high green reflectance, matching both bat and hummingbird visual systems. While hummingbird visitation remained stable across flowering seasons, bat visitation was highly variable, suggesting that *C. sellowiana* maintains a stable bimodal pollination strategy. Furthermore, pollen tube growth experiments showed that small amounts of cross‐pollen are sufficient to promote successful pollen tube development, even in the presence of self‐pollen. These findings reveal a strategy that may mitigate self‐pollen interference in a self‐incompatible species. Our results highlight the ecological importance of functional and temporal complementarity in bimodal pollination systems and underscore how mixed pollination strategies may enhance reproductive success and resilience in diverse pollinator environments.

## Graphical abstract

We investigated the reproductive biology of *Callianthe sellowiana*, a high‐altitude Atlantic Forest species with traits associated with both bat and hummingbird pollination. Our results show that both pollinator groups are equally effective, and reproductive success is enhanced when both are present, highlighting the functional and temporal complementarity of this stable bimodal system.

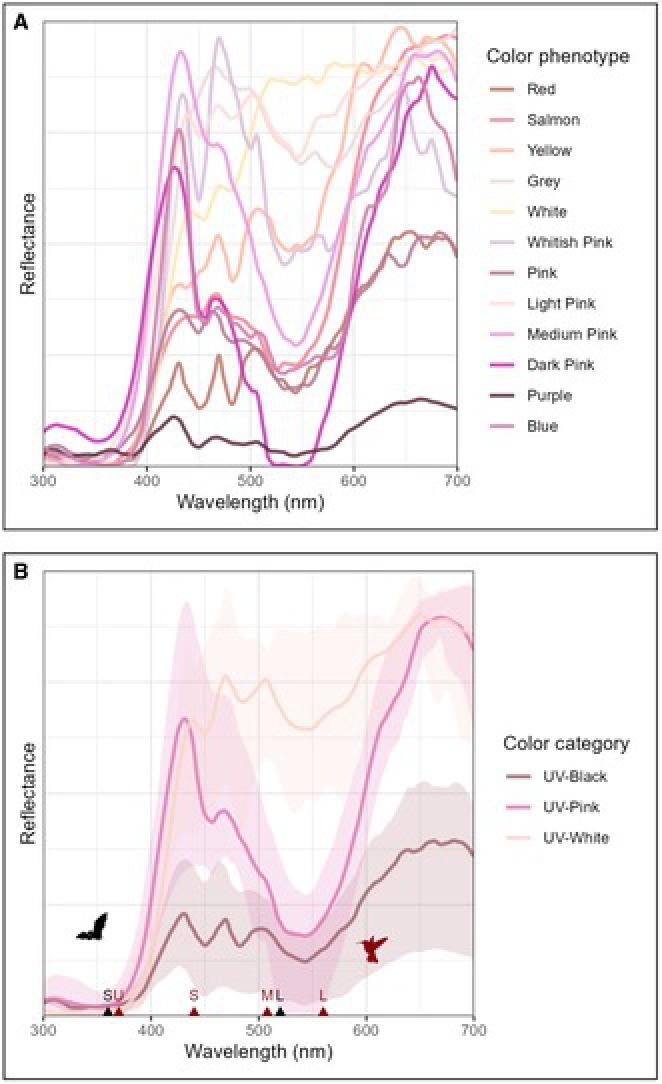

## 1 Introduction

Bimodal or mixed pollination systems are characterized by the presence of two unrelated groups of pollinators with significant effectiveness, while the plant species display flowers with intermediate adaptation between pollination syndromes (Manning and Goldblatt 2005; Rosas‐Guerrero et al. 2014). This stable pollination system can evolve if pollination by both functional groups results in an overall fitness gain (Aigner 2006; Barrionuevo et al. 2021). Nevertheless, the presence of two pollinator functional groups may also suggest an evolutionarily unstable state, potentially representing the initial stages of a pollination shift. The differences in effectiveness between different pollinators can lead to different results in plant reproductive success. In this context, bimodal systems can have two different pollinator groups contributing with nearly equal effectiveness, and systems with one most effective and a secondary less effective pollinator group (Waser et al. 1996; Manning and Goldblatt 2005). In these cases, the less effective pollinator is often the plant lineage's ancestral pollinator, which has become relatively ineffective (Rosas‐Guerrero et al. 2014).

This principle, which is central to pollination syndromes, suggests that floral traits evolve primarily in response to the most frequent and efficient pollinator group (Stebbins 1970; Faegri and van der Pijl 1979). Following the concept of pollination syndromes (sensu Faegri and van der Pijl 1979), plants present sets of convergent floral traits that have evolved in response to selection by specific pollinator groups and thus reflect the identity of the main floral visitors. These traits are often associated with the sensory system and foraging behavior of the species' pollinators, supporting the idea of “most effective pollinator” (Stebbins 1970). Although pollination syndromes have guided research and pollinator prediction for decades, they have been criticized because some floral traits (especially the quantitative ones: color, scent, and shape) are often evaluated using subjective or non‐standardized criteria, potentially leading to a misclassification of syndromes (Abrahamczyk et al. 2017). Furthermore, the application of this concept has been challenged by increasing evidence of generalized pollination systems, where floral traits do not match a single pollinator group, and species are visited by multiple functional groups (reviewed by Dellinger 2020). Still, pollination syndromes remain useful in certain cases, especially for vertebrate‐pollinated species (see also Rosas‐Guerrero et al. 2014).

Among vertebrate‐pollinated plants, those primarily pollinated by hummingbirds often exhibit flowers with a set of characteristics including tubular or narrowly shaped corollas, vivid colors, often red, orange, and pink, low or no scent production, and nectar positioned deeply within the floral tube (Coimbra et al. 2020). These traits function both to attract hummingbirds and to restrict access by less specialized floral visitors (Bergamo et al. 2016; Coimbra et al. 2020). However, some species with recorded hummingbird pollination possess characteristics that are not common to this pollination syndrome, such as crepuscular anthesis, the presence of odor, and the production of large volumes of diluted nectar. These characteristics are frequently attributed to bat pollination (Muchhala and Thomson 2010; Wanderley et al. 2020, Domingos‐Melo, Diniz, et al. 2020).

In many cases, chiropterophily has an evolutionary origin in ornithophily (Muchhala and Thomson 2010; Barreto et al. 2024). This evolutionary pathway is more often observed among New World bat‐pollinated species, and despite often being considered an evolutionary dead end (Fleming et al. 2009), there is also evidence of reversals from bat pollination, including some cases of bat‐to‐hummingbird pollination shifts (Fleming et al. 2009; Barreto et al. 2024). Such reversals are likely facilitated by the functional similarities between Glossophaginae and Lonchophyllinae bats and Trochilidae, including hovering flight, high metabolic demands, and long, specialized mouthparts, which may promote convergence in floral use across these groups. Regardless of evolutionary direction, floral traits associated with bat and hummingbird pollination may overlap in some species, such as the production of abundant nectar and scent (Muchhala and Thomson 2010; Barreto et al. 2024).

Such overlapping traits may reflect intermediate stages in a pollinator shift or result in functional generalization, where species are pollinated by more than one functional group (Barreto et al. 2024). Bat–hummingbird pollination can either have hummingbirds as the ancestral pollinator or be a bimodal system with adaptations to both bat and hummingbird pollinators (Dellinger, Scheer, et al. 2019). For the latter case, crucial features of bimodal systems are necessary, such as diurnal and nocturnal anthesis, morphological fit and attraction traits for both pollinators, as well as continuous nectar production as a reward (Muchhala et al. 2009; Queiroz et al. 2016). Additionally, due to the longer flight distances and larger pollen loads bats can carry, bat pollination is considered more effective when compared to pollination by hummingbirds (Fleming 2024, but see Abrahamczyk and Steudel 2022).

Although nectarivorous bats are primarily oriented by scent and echolocation (Gonzalez‐Terrazas et al. 2016), they are thought to use visual cues at dusk to detect freshly open flowers while they are still visible and then rely on spatial memory or acoustic cues to navigate flower resources (Domingos‐Melo et al. 2021). Since hummingbirds are primarily vision‐oriented, visual traits may be under selection by both agents in mixed systems, unlike scent. While hummingbird‐pollinated flowers are often red (Coimbra et al. 2020), those pollinated by bats are mostly white (Domingos‐Melo et al. 2021), or greenish (e.g., Machado et al. 1998; Sazima et al. 1999), neither presenting prominent UV reflection. Morphological matching such as corolla opening is also highly relevant for pollen transfer for both bats and hummingbirds (Muchhala 2007), without intermediate optima. Flowers with diurnal anthesis may also use depth as a signal to hummingbirds, since deeper flowers present higher rewards (Tavares et al. 2016). Among angiosperms, mixed pollination systems are frequently observed and often associated with pollinator niche partitioning (Dellinger, Artuso, et al. 2019; Dellinger, Scheer, et al. 2019; Lagomarsino and Muchhala 2019). Also, mixed pollination systems can be advantageous for plant species (e.g., Queiroz et al. 2016), potentially reducing the probability of local extinction and also maintaining reproductive success under low pollinator abundance (Barreto et al. 2024). In bat‐hummingbird pollination systems, for example, there is a temporal segregation with hummingbirds acting as diurnal pollinators and bats as nocturnal pollinators (Barreto et al. 2024). Divergent selection imposed by multiple pollinator guilds in mixed systems often promotes within‐species floral diversity, yielding both continuous variation and discrete polymorphisms (Sapir et al. 2021; Bergamo et al. 2016; Kellenberger et al. 2019; Wenzell et al. 2025). Such variation occurs not only among individuals but also within individuals, as flowers can shift shape (Van Doorn and Van Meeteren 2003; Kwiatkowska et al. 2019) and color (Brito et al. 2015) over the course of anthesis.

*Callianthe sellowiana* (Klotzsch) Donnell (Malvaceae) is a treelet species endemic to the Atlantic Forest of Rio de Janeiro, with flowers characterized by a three‐day anthesis period beginning at sunset, abundant nectar production, and emission of floral scent. Although species within this genus are typically pollinated by both bats and hummingbirds, the primary functional group contributing to the reproductive success of *C. sellowiana* remains unclear. Given the greater pollen transport capacity and longer flight distances of bats, when compared to hummingbirds, which often present territorial behavior hindering cross‐pollination, we hypothesize that these nocturnal pollinators play a more significant role in seed production compared to hummingbirds. However, considering the high visitation rates of hummingbirds in forest environments, it is possible that even a small amount of cross‐pollen deposition is sufficient to trigger fruit set, despite the possible self‐incompatibility of the species (as frequent in the genus; Buzato et al. 1994; Sazima et al. 2003; Wolowski et al. 2013).

To address these questions, we aim to (1) assess the relative contribution of diurnal and nocturnal pollinators to seed production in *C. sellowiana* and (2) determine whether there is a minimum threshold of cross‐pollen deposition required to initiate fruit and seed formation. Furthermore, we analyzed three traits that are potentially relevant to both bats and hummingbirds: (3) flower visual signals, (4) corolla opening, and (5) nectar production. Specifically, we test the hypotheses that (i) bats are more effective pollinators due to their ability to transport larger quantities and higher‐quality pollen and (ii) fruit set may occur even with limited cross‐pollen deposition, as hummingbirds, although not universally territorial, frequently exhibit this behavior, which can hinder cross‐pollen transfer dynamics. (iii) The visual signals of most floral color phenotypes are tuned to peak brightness at dusk, selected by bats (sensu Domingos‐Melo et al. 2021), since hummingbirds show no color preferences; (iv) corolla opening alternates across the diel cycle, wider at night and narrower by day, optimizing morphological matching to bats and hummingbirds across anthesis (sensu Muchhala 2007); and (v) nectar traits are intermediate between known cases of bat and hummingbird‐pollinated plants.

## 2 Materials and Methods

### 2.1 Study System

**FIGURE 1:**
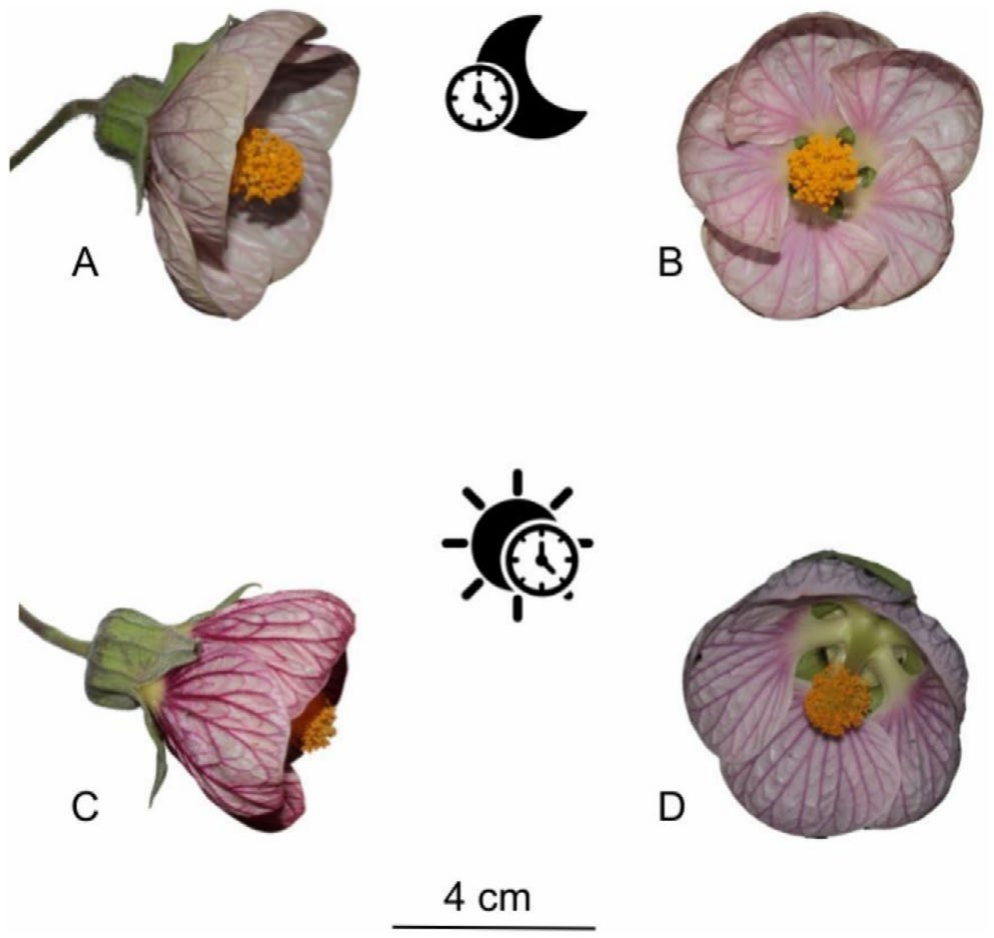
Floral variation in corolla opening throughout anthesis. (A) Lateral view, (B) frontal view of floral opening during the nocturnal period (bat visitation period), and (C) lateral view, and (D) lateral‐frontal view of corolla opening during the daytime period (hummingbird visitation period) in *Callianthe sellowiana* at Itatiaia National Park, Brazil.

In this study, we focused on the bimodal species *Callianthe sellowiana* (Klotzsch) Donnell in the dense ombrophilous montane forest (sensu Veloso et al. 1991) of the Itatiaia National Park (INP). In the study area, *C. sellowiana* occurs between 1800 and 2100 m a.s.l., blooming from April to August, peaking in May. Within the years of 2023 and 2024, for the first time, we observed a second blooming season between December and February. In the study population, we recorded 31 adult individuals, along with a higher number of seedlings distributed across the area. The flowers of the species display some overlap between ornithophilous and chiropterophilous characteristics. The species has flowers varying from white to deep purple, crepuscular anthesis, and abundant nectar production lasting throughout the day, with scent emission starting at dusk. The species consists of treelets (up to 5‐m tall) with campanulate flowers starting anthesis at the end of the day and remaining open for three and a half days. Across the anthesis period, the flowers differ in corolla opening, being narrower during the day and wider at nighttime (Figure 1). There are some records of bat pollination in the genus (Buzato et al. 1994, 2000), and two bat species were identified visiting the flowers of *Callianthe bedfordiana*: *Anoura caudifer* (E. Geoffroy, 1818) and *Anoura geoffroyi* (Gray 1838) (Canela 2006; Wolowski et al. 2013) at the study site. These records provide direct evidence that nectarivorous bats interact with species of the genus *Callianthe* in the same habitat, supporting the likelihood of their role as pollinators of *C. sellowiana*.

### 2.2 Corolla Opening Measurements

During a 4‐day assessment, we marked flowers in pre‐anthesis and measured corolla opening width (vertical and horizontal) for each marked flower using a digital caliper at 8:00–9:00 AM and 08:00–9:00 PM on the following days.

### 2.3 Nectar Measurements

To measure nectar volume and concentration, we took two approaches: with and without visitor exclusion. We bagged nine flower buds to exclude visitors before measurement, and in parallel selected nine freshly opened flowers to assess nectar standing crop, i.e., nectar available during exposure to floral visitors. All measurements were taken in the morning (08:00–09:00 AM) using a Hamilton 25 μL microsyringe and a hand‐held refractometer to estimate nectar volume and concentration per flower, respectively. Since only one of the bagged flowers opened during our expedition, we relied mostly on nine standing crop measurements, taking nectar in the morning from freshly opened flowers that had been exposed to visitors.

### 2.4 Pollen Tube Growth

We observed pollen tube growth to evaluate the reproductive system of *C. sellowiana*. We sampled 23 flowers and applied four pollination treatments with varying proportions of cross‐ and self‐pollen and a control: pure self‐pollen load (PS; *n* = 5 flowers), self‐pollen supplemented with pollen load from one cross‐pollinated donor (S1; *n* = 10), self‐pollen supplemented with pollen load from three cross‐pollinated donors (S3; *n* = 5), and control: natural pollination (NP; *n* = 5). The pure cross‐pollen treatment was not applied due to the position of the stigma, which is inserted into the androphore. We bagged the flowers of the individuals at the floral bud stage, and when in anthesis, we collected the flowers in the morning and applied the treatments. Anthers of the same flowers were removed and used for manual pollination following the treatments. For better pollen quality in the S3 treatment, we used three anthers from three different individuals. We then maintained the flowers in agar gel for 24 h to observe pollen tube development. In the laboratory, we assessed pollen tube growth using Martin (1959). For this, we softened the pistil samples in NaOH and, when necessary, clarified them with NaClO. We stained the pistils with 1% aniline blue and mounted them on slides for observation under a fluorescence microscope. We counted the number of conspecific pollen grains adhered to the stigma and the number of pollen tubes growing in the style. Additionally, we counted the number of pollen grains that germinated but exhibited anomalies or had their growth interrupted in the stigma region (Maruyama et al. 2016).

### 2.5 Color Signals and Flower Color Categorization

For the analysis of color signals, which, unlike olfactory signals, are relevant for both hummingbird (Stiles 1976; Goldsmith and Goldsmith 1979) and bat pollination (Domingos‐Melo et al. 2021), we sampled 12 different‐colored flowers (*n* = 12 individuals, one flower per individual) of *C. sellowiana*, naming them arbitrarily according to human vision and registering the number of reproductive individuals with similar color phenotypes in the population. We then took 4–15 reflectance measurements of each flower using a portable spectrometer (USB 4000; Ocean Optics) at an angle of 45°. We used barium sulfate (BaSO_4_) as the white standard and a black chamber as the black standard (Bergamo et al. 2016; Coimbra et al. 2020). We restricted the analyses to the 300–700 nm wavelength range, which falls under the spectral sensitivity of animal pollinators. Since flower color variation is continuous in this species and each phenotype presents a unique reflectance, we named each of the 12 individual phenotypes arbitrarily and then objectively categorized their spectra into discrete color categories following Coimbra et al. 2020, according to their relative reflectance at the UV, blue, green, and red wavelength bands. We used the following absorption/reflection thresholds: 20% for the UV band, 30% for blue, 40% for green, and 60% for red, averaging all spectral measurements for each phenotype before categorizing their color signals. We then estimated the relative abundance of each color category in the studied population by multiplying the number of reproductive individuals with a given phenotype by the number of phenotypes in each color category.

### 2.6 Visual Modeling

To analyze how different color categories may attract bats and hummingbirds under mixed pollination selection, and test the hypothesis that phenotypes attractive to both are most abundant in the population, we took two approaches. For hummingbirds, whose visual system is already known and corroborated by experimental tests, we used flower chromatic contrasts against the background (CCB) as a proxy for flower conspicuousness to hummingbird vision using the spectral sensitivity of a model hummingbird species, *Sephanoides sephaniodes* (Lesson, 1827): u: 370; s: 440; m: 508; and l: 560 (Herrera et al. 2008) and the receptor noise model of Vorobyev and Osorio (1998) under the R‐package *pavo* (Maia et al. 2019). In this metric, higher CCB values indicate individuals with flowers that are more easily distinguishable from the leaf background. Similarly, achromatic contrast against the background (ACB) can aid in long‐distance detection and low‐light conditions. We used the average leaf reflectances of Coimbra et al. 2020 as the standard leaf background and “forest shade” illuminant in the *vismodel*() and *coldist*() functions. For phyllostomid bats, whose visual sensitivity is known but which have not gone through behavioral validation, we used mean intensity of reflection at the UV and green bands as proxies for conspicuousness to bats at mesopic conditions (at dusk). Since their spectral sensitivities peak in the ultraviolet (s: 365 nm) and green (l: 520 nm, Müller et al. 2009), we considered these two bands of the spectrum as meaningful to their visual systems.

### 2.7 Floral Visitors' Observations

A total of 53 h (23 h at daytime and 28 h at nighttime) of direct focal observations were conducted in the years of 2022 (40 h:20 h diurnal and 20 h nocturnal) and 2023 (13 h:5 h diurnal and 8 h nocturnal) distributed across 20 days during four field expeditions. Observations were made without the use of camera traps; instead, visits were recorded in situ by the observer, aided by photographic records for later identification of visitors. For diurnal visitors, observations were carried out between 7:00 AM and 5:00 PM, with a minimum period of one and a half hours for each focal individual. For nocturnal visitors, observations occurred from 5:00 PM to 5:00 AM, following the same interval pattern between focal individuals. Observations included all open flowers of the selected individuals, with focal monitoring alternating among flowering plants used in the pollinator exclusion treatments. Observed visitors were classified according to their behavior on the flower as: (i) pollinator—if it had contact with the stigma and carried pollen grains adhered to an appropriate body part; and (ii) theft—visited with collection of pollen and/or nectar, without contact with the reproductive parts.

### 2.8 Pollinator Exclusion

To isolate the effects of different pollinators on the studied species, we conducted a pollinator exclusion experiment. For *C. sellowiana*, 16 individuals were selected along the road to the upper part of the INP. Three treatments were applied during the flowering period of the species: nocturnal exclusion (NE, *n* = 69 flowers), diurnal exclusion (DE, *n* = 69), and control (C, *n* = 73) on each individual. For the NE treatment, floral buds were bagged at twilight to exclude pollinators, and in the following morning, the bags were removed, allowing access for diurnal visitors. For the DE treatment, flowers were left accessible overnight and bagged in the next morning before the diurnal pollinator activity period. As anthesis lasts three and a half days, for both exclusion treatments we bagged the flowers and unbagged them throughout their anthesis time. In the control treatment (C), flowers remained accessible to both diurnal and nocturnal pollinators without manipulation. Fruit set and seed production were quantified for each treatment a month after the end of the exclusion experiments. In total, 211 flowers from 16 individuals were included across all treatments.

### 2.9 Statistical Analysis

For corolla opening, we summarized each anthesis stage (Night 1, Day 1, Night 2, Day 2) with the across‐flower mean and its 95% CI, computed as mean ± 1.96 × SE (SE = SD/√*n*). Sample size (*n*) at each stage reflects the number of flowers with a non‐missing measurement at that stage only. To describe diel change within flowers, we calculated a paired night–day difference for each flower (Night—Day) per available cycle and, when two cycles were present, used the per‐flower average of those differences; we then reported the mean of these per‐flower differences with a 95% CI based on the SE of the paired differences. For nectar, we computed the mean ± SE of volume for bagged buds (visitor‐excluded) and for standing crop (exposed flowers). Nectar concentration (% sucrose equivalents) was summarized as mean ± SE using only flowers with volume > 0. To evaluate the pollen tube growth in *C. sellowiana*, we fitted a generalized linear mixed model with a negative binomial error distribution and a zero‐inflation term. The model included pollination treatment, pistil position (stigma, middle, and base of the style), and their interaction as fixed effects, while individual flowers were included as a random effect. Model assumptions were assessed graphically using the DHARMa package (Hartig 2022). Since we were specifically interested in comparing treatment effects among them, we performed a post hoc test using the package *emmean*s (Lenth et al. 2025). To compare visual parameters (derived from floral reflectance spectra and visual modeling) across color phenotypes, we used ANOVA models and post hoc Tukey's tests, checking model assumptions visually with QQ‐plots.

To assess the relative frequency of pollinators, we fitted a linear model (Gaussian distribution) using the R package *lme4* (Bates et al. 2015), with the proportion of visits by each pollinator species as the response variable. Visitation frequency per observation hour was calculated for each pollinator species. To compare visitation rates among species, we fitted a generalized linear mixed model (GLMM) with pollinator species as a fixed factor and observation day as a random factor, using a Gaussian error distribution. Pairwise differences between species were assessed using estimated marginal means (EMMs) with Tukey's adjustment for multiple comparisons. All analyses were performed in R (R Core Team 2020) using the packages lme4 and emmeans. For the pollinator exclusion treatment, we fitted two GLMMs using the function g*lmer* of the same package. In the first model, fruit set (bimodal response: fruit present = 1, fruit absence = 0) was the response variable, and in the second model, seed set (count response) was the response variable, modeled with Poisson distribution. In both cases, treatment (C, DE, and NE) was included as a fixed effect and individuals as a random effect. We checked all model assumptions graphically with the package *DHARMa* (Hartig 2022).

## 3 Results

*Callianthe sellowiana* presents terminal bell‐shaped flowers varying in color among individuals and from narrower to wider according to their anthesis stage. The scent is prominent at dusk and reminds of cabbage.

**FIGURE 2:**
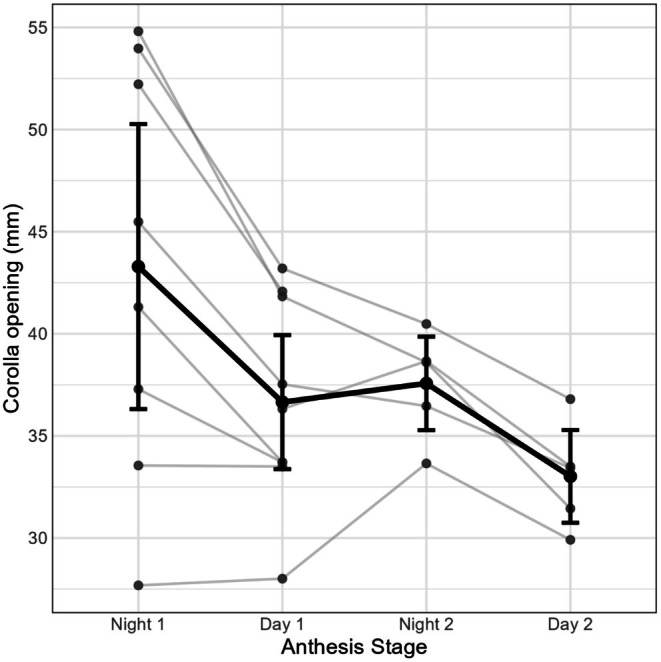
Corolla opening across anthesis stages in *Callianthe sellowiana*. Thin gray lines and points show individual flowers tracked through time (night: 20:00–21:00 h; day: 8:00–9:00 h). The thick black line and circles give the across‐flower mean at each stage, with vertical error bars indicating the 95% confidence interval (mean ± 1.96 × standard error).

Across two diel cycles (*n* = 9 flowers), corolla opening was greater at night than during the day (Table S1, Figures 1 and 2). Within‐flower averages indicated a mean night–day difference of 5.5 mm (95% CI: 3.2–7.8; *n* = 8).

As for nectar, only one bagged bud opened during our sampling, yielding 114.0 μL at 15% sugar. For standing crop, mean volume was 27.0 ± 16.6 μL (*n* = 9), and mean concentration, calculated from the subset with measurable nectar, was 21% ± 8% SE (*n* = 5).

**FIGURE 3:**
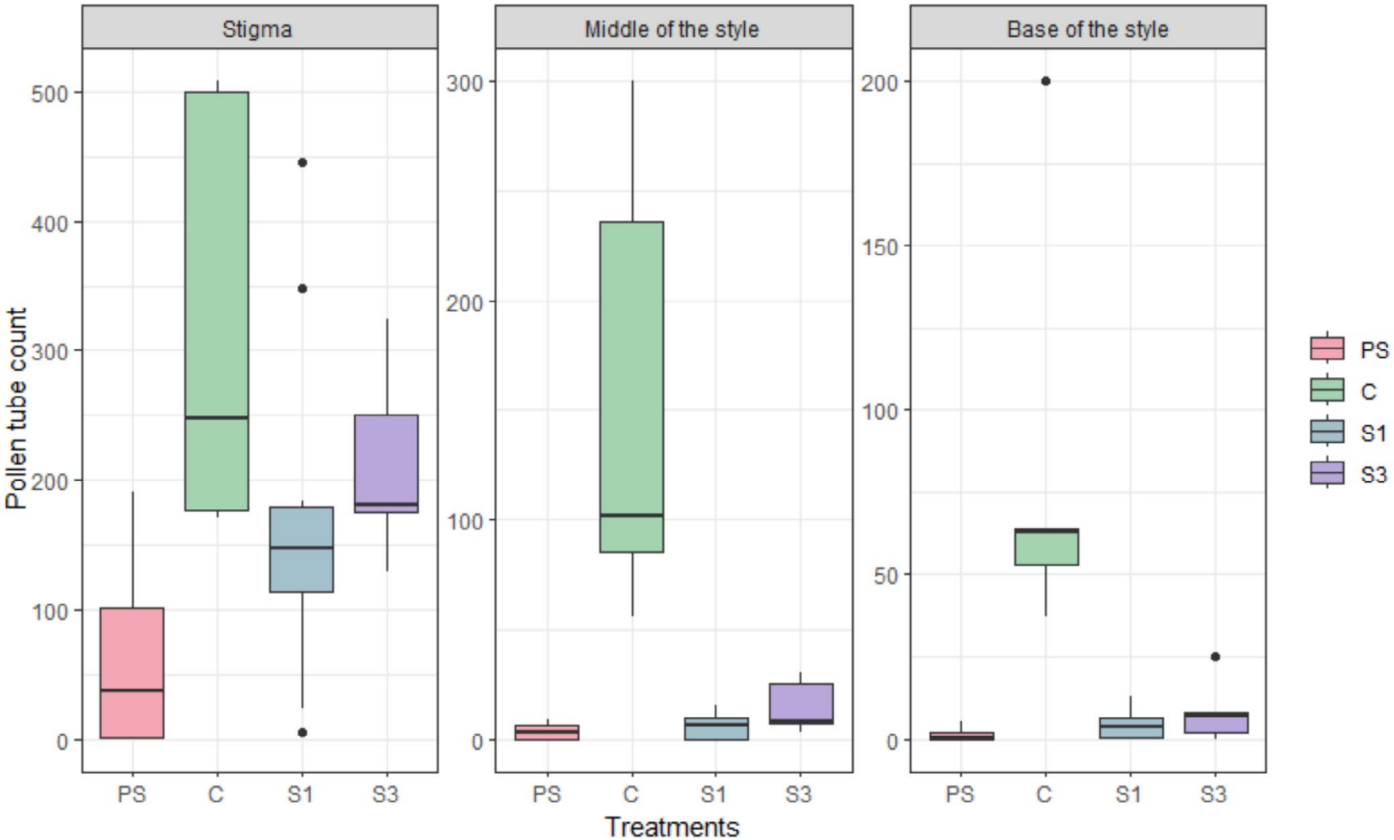
Pollen tube development in *Callianthe sellowiana* under different pollination treatments. Boxplots show the number of pollen tubes recorded at the stigma (left), middle of the style (center), and base of the style (right). Treatments: PS = autonomous self‐pollination; C = open‐pollination control; S1 = self‐pollen mixed with pollen from one cross donor; S3 = self‐pollen mixed with pollen from three cross donors. Boxes represent the interquartile range (IQR), horizontal lines indicate medians, whiskers extend to 1.5× IQR, and dots denote outliers.

Pollen tube germination confirmed self‐incompatibility in *C. sellowiana* (Figure 3). At the stigma, no significant differences were detected among all treatments (*p* > 0.1). At the tip of the style, the control treatment presented higher pollen tube counts compared to auto (EMM = 4.94 ± 0.41 vs. 0.83 ± 0.78; *p* < 0.0001), cross (1.85 ± 0.41; *p* < 0.0001), and mix (2.59 ± 0.43; *p* = 0.0004). At the base of the style, a similar pattern was observed, with the control showing significantly higher values than auto (4.34 ± 0.41 vs. –0.01 ± 0.81; *p* < 0.0001), cross (1.64 ± 0.43; *p* < 0.0001), and mix (2.08 ± 0.49; *p* = 0.0022). No other pairwise comparisons showed statistical significance (Table 1).

**TABLE 1:** Contrasts between treatments with different pollen loads.

Predictor variable	χ^2^	df	*p*
**ANOVA**			
Treatments	32.30	3	< 0.001
Position	143.74	2	< 0.001
Treatment * Position	25.61	6	< 0.001

Treatments	Estimate ± SE	*z*	*p*
**Contrast—Stigma**			
PS–NP	−1.975 ± 0.882	−2.239	0.113
PS–S1	−1.277 ± 0.769	−1.659	0.345
PS–S3	−1.635 ± 0.901	−1.814	0.267
NP–S1	0.698 ± 0.518	1.348	0.532
NP–S3	0.340 ± 0.574	0.591	0.935
S1–S3	−0.358 ± 0.526	−0.681	0.905
**Contrast – Middle of the style**			
PS–NP	−4.103 ± 0.861	−4.763	**< 0.0001**
PS–S1	−1.014 ± 0.867	−1.169	0.647
PS–S3	−1.760 ± 0.868	−2.027	0.178
NP–S1	3.090 ± 0.583	5.298	**< 0.0001**
NP–S3	2.344 ± 0.592	3.957	**< 0.001**
S1–S3	−0.746 ± 0.599	−1.247	0.597
**Contrast – Base of the style**			
PS–NP	−4.347 ± 0.902	−4.818	**< 0.0001**
PS–S1	−1.652 ± 0.959	−1.724	0.311
PS–S3	−2.087 ± 0.921	−2.265	0.106
NP–S1	2.694 ± 0.599	4.495	**< 0.0001**
NP–S3	2.259 ± 0.637	3.550	**0.002**
S1–S3	−0.435 ± 0.648	−0.671	0.908

*Note:* Bold values indicate significant effects at *p* < 0.05.

Abbreviations: Auto, pure self‐pollen load; Cross, self‐pollen with cross‐pollen from three different individuals; EMM, estimated marginal means; Mix, self‐pollen with cross‐pollen from one individual; SE, standard error.

**FIGURE 4:**
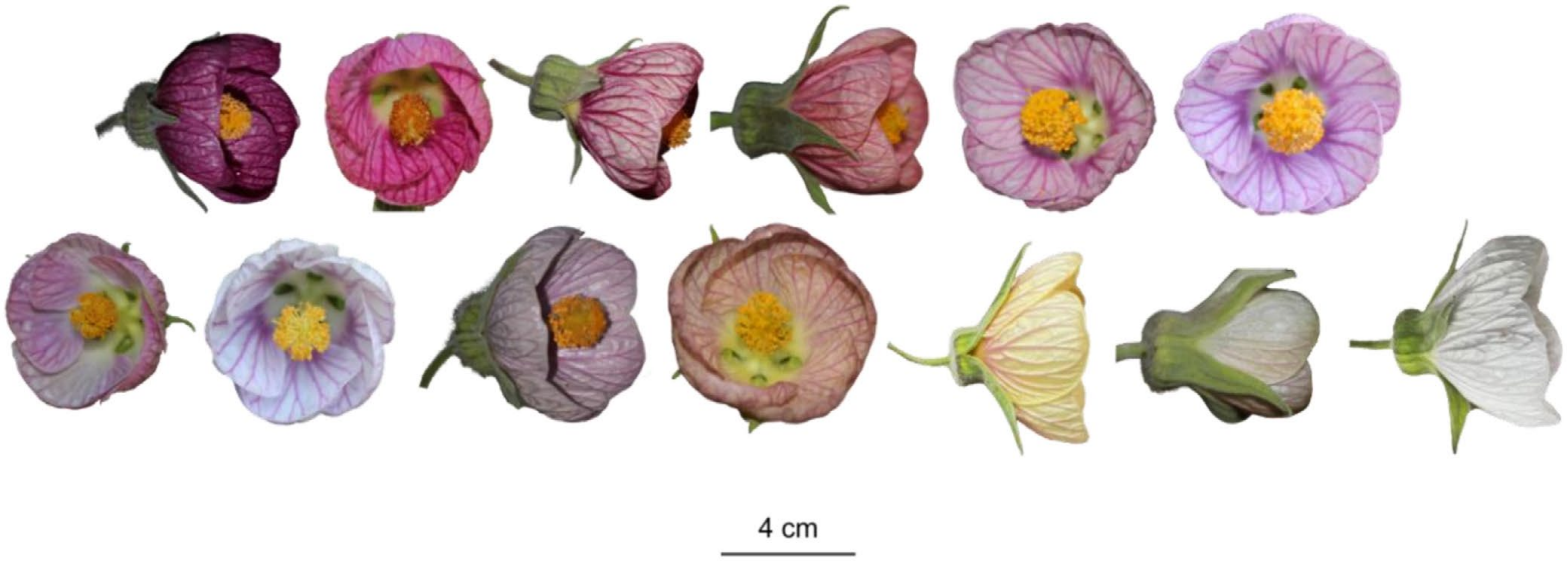
Floral color variation in *Callianthe sellowiana*. Flowers vary widely in pigmentation, ranging from deep purple to white. All color phenotypes exhibit prominent venation and bell‐shaped corollas. Scale bar = 4 cm.

**FIGURE 5:**
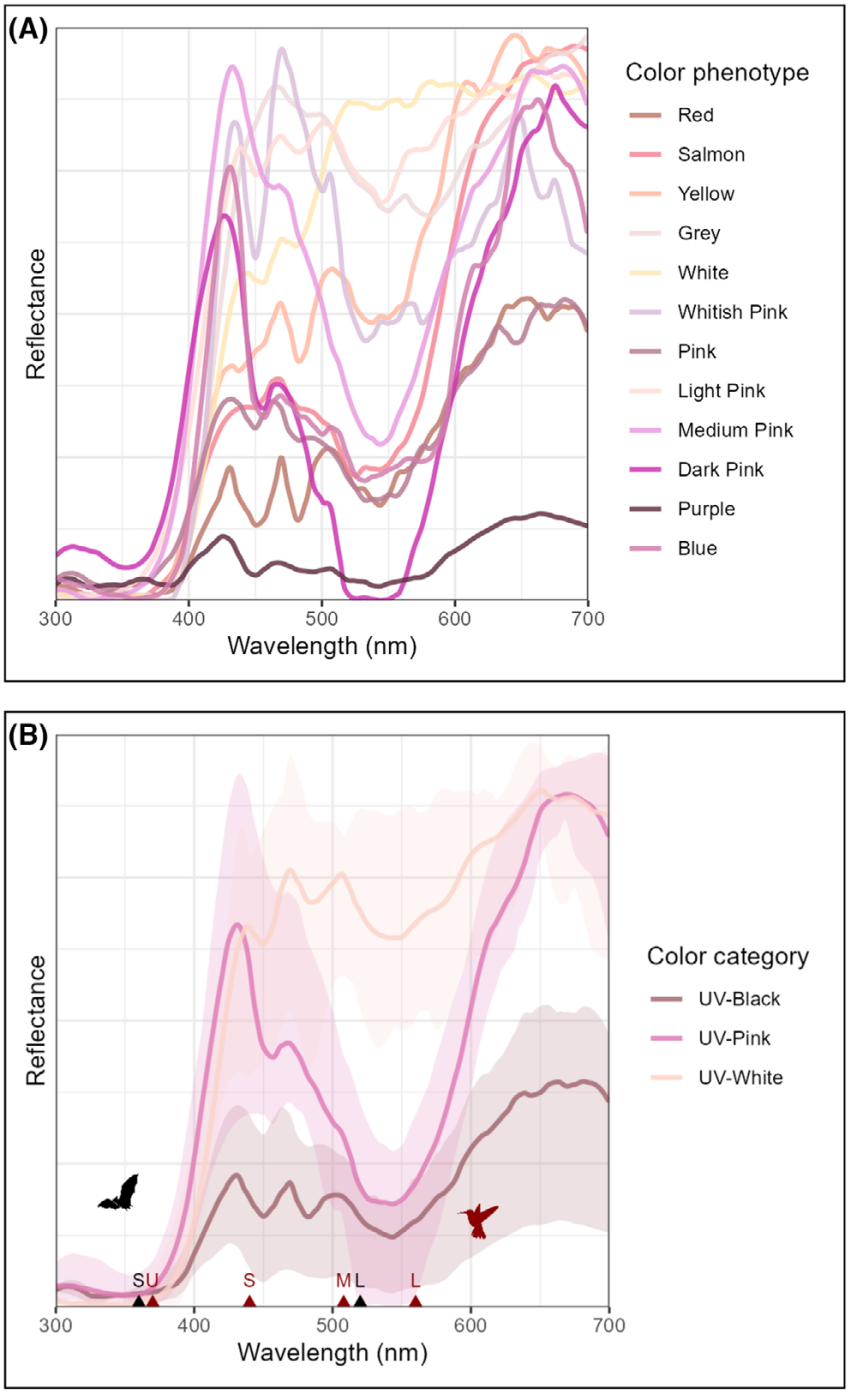
Mean flower reflectance for (A) 12 different‐colored individuals, i.e., phenothypes, of *Callianthe sellowiana* sampled; (B) Color categories. Spectral lines are colored according to their equivalent in human vision. In black, the spectral sensitivity of phyllostomid bats under mesopic conditions is shown (Müller et al. 2009); in red, the sensitivities of hummingbirds (Herrera et al. 2008). Letters indicate photoreceptor names (u: Ultraviolet sensitive; s: Short‐wavelength sensitive; m: Medium‐wavelength sensitive; and l: Long‐wavelength sensitive). Curves are colored according to their RGB approximates in human vision.

As for flower color, *C. sellowiana* presents high variation among individuals (Figure 4), with prominent darker venation in most color variations. Flower color for each individual may be anywhere in a spectrum ranging from white to deep purple, such as fuchsia, pinkish, lilac, or light yellow. Spectral analyses revealed only three constant features: low ultraviolet reflectance (0%–2%), peak reflectance at red wavelengths, and considerable intensity in the green range (Figure 5A). Using the method for objective color categorization of Coimbra et al. (2020), we were able to identify three groups of phenotypes (Figure 5B, Table 2, Table S2): UV‐absorbing white individuals (which reflect in all wavebands except UV, hereafter “UV‐White”), accounting for 42% of the population, followed by UV‐absorbing pink individuals (blue and red reflecting, hereafter “UV‐Pink”), with 32%, and UV‐absorbing black individuals (reflecting minimally in all wavebands, hereafter “UV‐Black”), making up 25% of the population.

**TABLE 2:** Category‐level summaries of floral color.

Color	*N*	UV	Green	CCB	ACB
UV‐Black	8	0.03 ± 0.02	0.17 ± 0.11	2.75 ± 2.19	0.41 ± 0.16
UV‐Pink	10	0.05 ± 0.04	0.27 ± 0.13	1.65 ± 0.25	0.23 ± 0.14
UV‐White	13	0.02 ± 0.02	0.70 ± 0.14	1.30 ± 0.06	0.19 ± 0.11

*Note:* Values are mean ± SD across phenotypes within each color category. *N* = total number of reproductive individuals (abundance) summed across phenotypes in the category. UV and Green = mean reflectance in the 300–400 nm and 500–600 nm bands, respectively. CCB = chromatic contrast to a leaf background (just‐noticeable differences, JND) under a *Sephanoides* visual model; ACB = achromatic (luminance) contrast to leaf (JND). Higher JND indicates greater visual distinguishability from the background.

Considering mean visual metrics for the three color categories, they only differed in green reflectance (*F* = 20, *p* < 0.001, Tables S3 and S4), where UV‐White, the dominant category, presents the most visually conspicuous flowers in bat vision (Table 2), while UV‐Pink and UV‐Black flowers probably pose challenges for visual detection by bats. Contrasts in hummingbird vision did not differ for color categories (Tables S3, S4), indicating they are equally conspicuous to birds, despite higher trends for UV‐Black flowers (Table 2).

**FIGURE 6:**
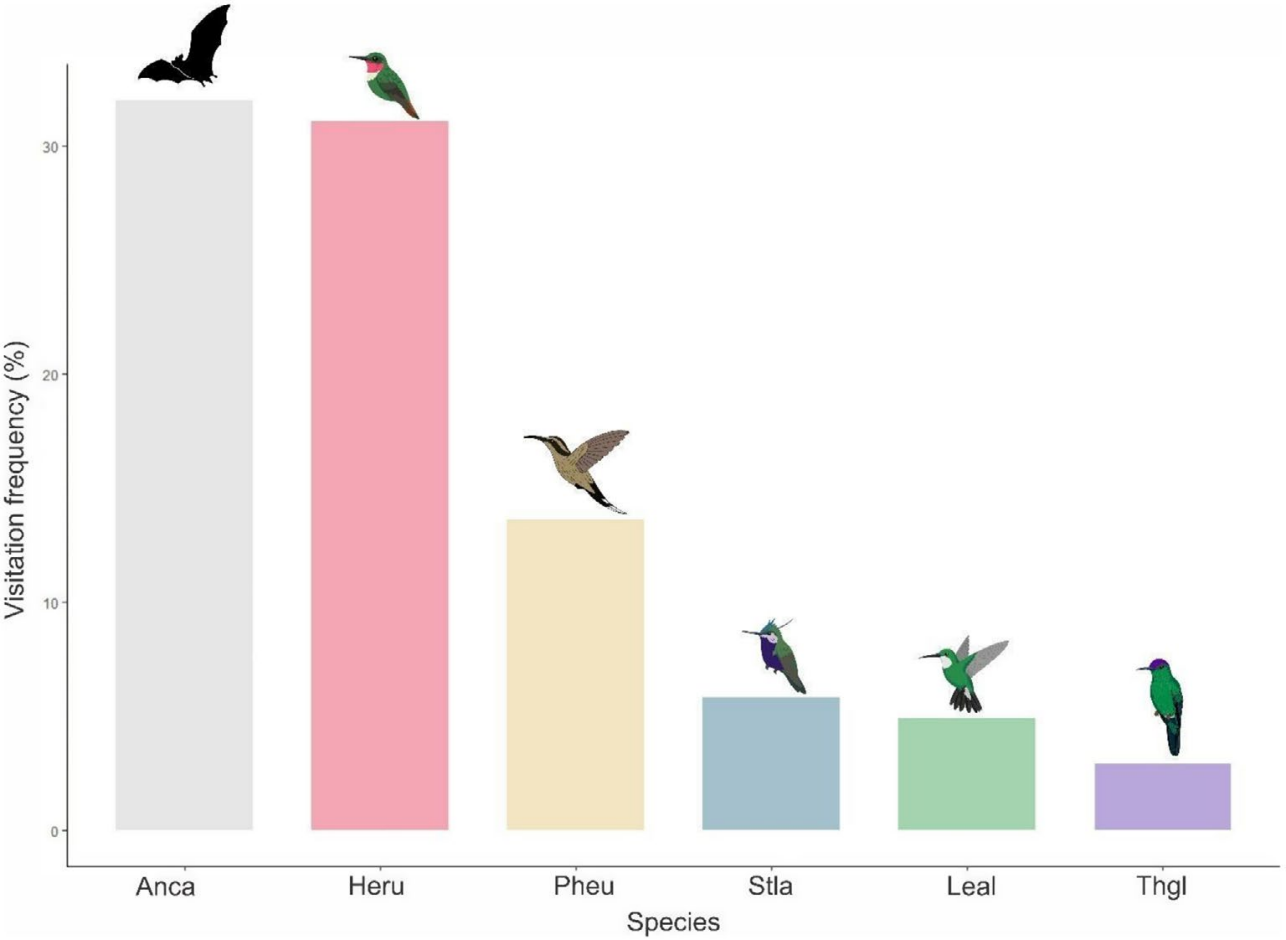
Relative visitation frequency (%) of two functional groups of pollinators. One bat species and five hummingbird species that pollinate *Callianthe sellowiana* at Itatiaia National Park, Brazil. Values are based on 53 h of focal floral observations conducted across two flowering seasons. Species abbreviations: Anca: *Anoura* cf. *caudiffer* (E. Geoffroy, 1818); Heru: *Heliodoxa rubricauda* (Boddaert, 1783); Pheu: *Phaetornis eurynome* (Lesson, 1832); Stla: *Stephanoxis lalandi* (Vieillot, 1818); Leal: *Leucochloris albicollis* (Vieillot, 1818); Thgl: *Thalurania glaucopis* (Gmelin, 1788).

**FIGURE 7:**
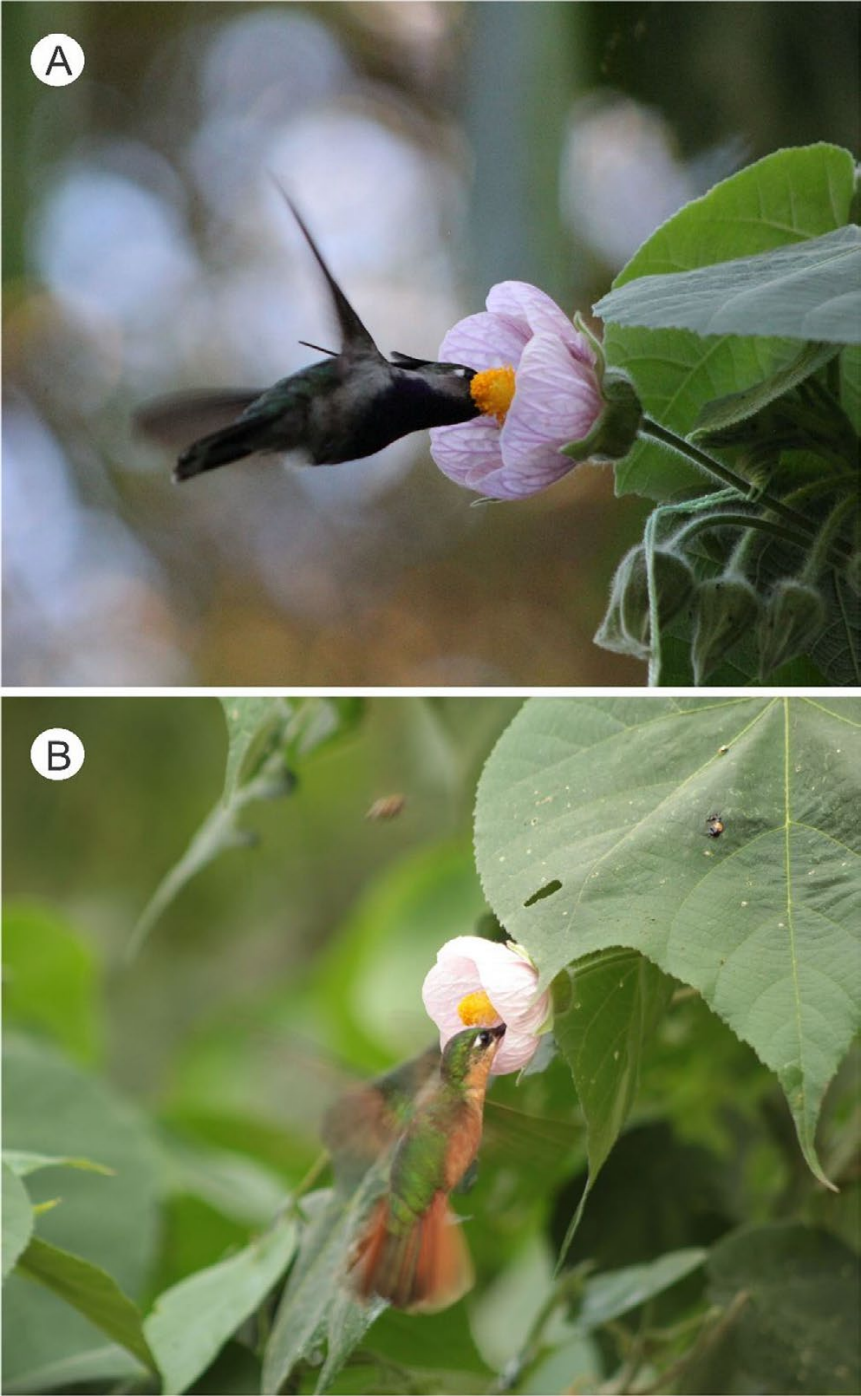
Hummingbird pollinators visiting *Callianthe sellowiana* flowers during diurnal anthesis at Itatiaia National Park, Brazil. A male of *Stephanoxis lalandi* (A) and a female of *Heliodoxa rubricauda* (B) accessing floral nectar and contacting the reproductive structures. Both species were recorded as effective pollinators in the studied population.

Five hummingbird species and one bat species were recorded as pollinators, varying in visitation relative frequency (%, Figure 6): *Stephanoxis lalandi* (Vieillot, 1818) (Figure 7A), *Heliodoxa rubricauda* (Boddaert, 1783) (Figure 7B), *Phaethornis eurynome* (Lesson, 1832), *Leucochloris albicolis* (Vieillot, 1818), *Thalurania glaucopis* (Gmelin, 1788), and *Anoura* cf. *caudifer*. Pairwise comparisons revealed that *Anoura* cf. *caudifer* had a significantly higher visitation frequency per hour than *Heliodoxa rubricauda* (EMM = 0.625, SE = 0.319, *p* = 0.0142), *Leucochloris albicollis* (EMM = 0.333, SE = 0.582, *p* = 0.0207), and *Phaethornis eurynome* (EMM = 0.700, SE = 0.450, *p* = 0.0267). No significant differences were found between *Anoura* cf. *caudifer* and *Stephanoxis lalandi* (EMM = 0.750, SE = 0.712, *p* = 0.1538) or *Thalurania glaucopis* (EMM = 0.750, SE = 1.007, *p* = 0.4302). Among hummingbirds, no pairwise comparisons showed significant differences in visitation frequency (all *p* > 0.90, Table 3). No nocturnal visitors were observed during the blooming period in 2022, despite fruit set occurring in the DE treatments (Table S5). Fruit set and seed production did not differ between DE and NE exclusion treatments (*β* = −20.3 ± 19.8, *p* = 0.56), and the C treatment was the only one with a statistical difference, between both DE (*β* = 85.1 ± 19.8, *p* < 0.001) and NE (*β* = 64.8 ± 19, *p* < 0.01) treatments (Table 4).

**TABLE 3:** Contrasts between pollinator species in hourly visitation frequency to *Callianthe sellowiana* in Itatiaia National Park, Brazil.

Contrast	EMM ± SE	df	*p*
*Anoura* – *Heliodoxa rubricauda*	1.44 ± 0.38	18.4	**0.0142**
*Anoura* – *Leucochloris albicollis*	1.88 ± 0.52	16.9	**0.0207**
*Anoura* – *Phaethornis eurynome*	1.56 ± 0.45	19.0	**0.0267**
*Anoura* – *Stephanoxis lalandi*	1.50 ± 0.58	19.0	0.1538
*Anoura* – *Thalurania glaucopis*	1.50 ± 0.78	15.3	0.4302
*Heliodoxa rubricauda* – *Leucochloris albicollis*	0.44 ± 0.45	18.3	0.9215
*Heliodoxa rubricauda* – *Phaethornis eurynome*	0.11 ± 0.35	15.3	0.9994
*Heliodoxa rubricauda* – *Stephanoxis lalandi*	0.06 ± 0.52	16.3	1.0000
*Heliodoxa rubricauda* – *Thalurania glaucopis*	0.06 ± 0.77	18.1	1.0000
*Leucochloris albicollis* – *Phaethornis eurynome*	−0.32 ± 0.49	13.8	0.9829
*Leucochloris albicollis* – *Stephanoxis lalandi*	−0.38 ± 0.63	19.0	0.9896
*Leucochloris albicollis* – *Thalurania glaucopis*	−0.38 ± 0.87	19.0	0.9976
*Phaethornis eurynome* – *Stephanoxis lalandi*	−0.06 ± 0.58	19.0	1.0000
*Phaethornis eurynome* – *Thalurania glaucopis*	−0.06 ± 0.82	19.0	1.0000
*Stephanoxis lalandi* – *Thalurania glaucopis*	0.00 ± 0.91	19.0	1.0000

*Note:* Pollinators included five hummingbird species ( *Stephanoxis lalandi* , *Heliodoxa rubricauda*, *Phaethornis eurynome* , *Leucochloris albicollis* and *Thalurania glaucopis* ) and one bat species (*Anoura cf. caudifer*). Bold values indicate significant effects at *p* < 0.05.

Abbreviations: EMM, estimated marginal means; SE, standard error.

**TABLE 4:** Result of the analysis of variance (ANOVA) from the Generalized Linear Mixed Model comparing seed formation among pollinator exclusion treatments conducted on *Callianthe sellowiana* in Itatiaia National Park.

Predictor variable	χ^2^	df	*p*
**ANOVA**			
Treatments	20.1	2	< 0.0001

Treatments	Estimate ± SE	*t*	*p*
**Contrast**			
C—DE	85.1 ± 19.8	4.297	< 0.001
C—NE	64.8 ± 19.8	3.271	< 0.01
DE—NE	−20.3 ± 19.8	−1.026	0.56

*Note:* df, degrees of freedom; Estimate, regression estimate; *t*, *t*‐test value of the regression coefficients; χ^2^, sum of squares. Treatments: C, control; DE, diurnal exclusion; NE, nocturnal exclusion.

## 4 Discussion

Pollinator exclusion treatments indicated that reproductive success in *Callianthe sellowiana* was higher when both bats and hummingbirds contributed to pollination, rather than when only one functional group was acting as a pollinator. The temporal segregation in pollinator activity (i.e., hummingbirds during the day and bats at night) promotes temporal niche partitioning (Dellinger, Artuso, et al. 2019; Dellinger, Scheer, et al. 2019; Lagomarsino and Muchhala 2019), allowing pollination to occur throughout the anthesis period, a pattern previously observed in other bimodal pollination systems (Cárdenas‐Calle et al. 2020). This functional complementarity enhances reproductive success, as flowers benefit from contributions by both pollinator groups (Blüthgen and Klein 2011). Temporal complementarity is commonly reported in diurnal and nocturnal pollination systems (Fleming et al. 2001; Cárdenas‐Calle et al. 2020, but see Dellinger, Scheer, et al. 2019). In *C. sellowiana*, functional complementarity is facilitated by dynamic floral morphology, which exhibits a narrower corolla diameter during the day and a wider diameter at night than other species in the genus (Buzato et al. 2000). Such morphological changes may promote effective contact between pollinators and reproductive structures, in contrast to previously reported cases where floral morphology excluded one pollinator group (Muchhala 2003). Although no study has explicitly associated photonastic movements with mixed pollination systems, analogous mechanisms illustrate how floral movements can mediate pollination outcomes. In *Viola banksii*, nyctinastic closure alternates between cross‐ and self‐pollination (Kwiatkowska et al. 2019), while in *Silene dioica* , diurnal and nocturnal pollinators impose contrasting selective pressures on floral traits (Barreto et al. 2024). Together, these cases suggest that temporal shifts in floral morphology, including photonastic responses, may provide a flexible pathway to accommodate distinct pollinators without requiring major morphological change.

Throughout the study, *C. sellowiana* exhibited two distinct flowering periods. The first occurred in June, during the dry and cold season. During this period, no bat visits were recorded. The second period was in February, corresponding to the rainy season. In this period, bat visitation increased notably, except during the full moon period (I. Paglia, pers. obs.). The pattern of *Anoura* cf. *caudifer* differed significantly only from the hummingbird species with higher relative visitation frequencies, but not from the less frequent ones, likely reflecting the high day‐to‐day variation in bat activity. Although *Anoura* reached high visitation peaks on certain nights, it was absent on many others, reducing the overall consistency of its visitation rate and resulting in no significant differences compared to the least frequent hummingbird species. Due to marked fluctuations in bat visits, it is unlikely that bats exert a consistent selective pressure on floral traits, reducing the possibility of a shift toward exclusive bat pollination. Nonetheless, the maintenance of floral traits that match with both pollinators and the prevalence of hummingbird–bat pollination systems in *Callianthe* species (Buzato et al. 1994; Sazima et al. 2003) supports the hypothesis of a stable bimodal strategy. Hummingbird visitation, in contrast, was consistent in both flowering periods. The diverse ornithophilous flora at the study area could create a competitive environment for floral visitors. *C. sellowiana* has a low local abundance, with 31 reproductive individuals found in our sampling, and less abundant species may be at a disadvantage in competitive pollination networks, where dominant species can monopolize pollinators (Bergamo et al. 2022). In this scenario, a bimodal pollination strategy may reduce the negative effects of interspecific competition, ensuring reproductive success through functional complementary mechanisms.

Floral color variation was pronounced within the studied population, with individuals bearing flowers that ranged from white to deep purple. This type of intra‐population variation has also been reported in other bimodal pollination systems (e.g., Bergamo et al. 2016; Kellenberger et al. 2019), and may result from divergent but simultaneous preferences imposed by the two pollinator groups. For instance, Muchhala (2003) experimentally tested whether bats and hummingbirds exhibited color preferences and whether floral color affected reproductive success; the study found no significant differences in visitation rates or reproductive success among the different color phenotypes. Although we were unable to test this in *C. sellowiana* due to logistical limitations in the field, it is likely that a similar pattern occurs in this species. Despite the noticeable color variation, all floral phenotypes exhibited minimal to no UV reflectance and showed high reflectance in the red wavelength range (~600–700 nm), which is common for hummingbird‐pollinated flowers (Coimbra et al. 2020), except for some absolute‐white flowers (Lunau et al. 2011).

Most color phenotypes present inflection points, i.e., parts of rapidly changing reflectance optimal for detection, clustering around 400 and 500 nm. At 400 nm, these loosely match both bat (s: 365 nm) and hummingbird (u: 370 nm) short‐wavelength sensitive photoreceptors; at 500 nm, the same happens for both bat (l: 520) and hummingbird (m: 508) green photoreceptors. The combination of low UV reflectance and relatively high reflectance in the green spectrum found for most different‐colored individuals likely makes flowers quite visible to bats, which possess dichromatic vision and rely primarily on achromatic rather than chromatic cues (Winter et al. 2003; Müller et al. 2009) when foraging for flowers at dusk (Domingos‐Melo et al. 2021). Green‐absorbing phenotypes such as UV‐Pink and UV‐Black might still be detected through scent and echolocation, and possibly slightly preferred by hummingbirds conditioned to darker‐colored flowers.

Therefore, floral color variation in *C. sellowiana* does not appear to be under significant pollinator selection, as both hummingbirds and bats can detect and are attracted to the flowers, regardless of flower color. This is corroborated by our findings of similar visual conspicuousness to both bats and hummingbirds among individuals, suggesting little to no selective pressure on this trait by either bats or hummingbirds, which may sustain the color variation of this species (Buzato et al. 1994, *sensu* Sapir et al. 2021). Also, since *C. sellowiana* is self‐incompatible and pollen flows among color phenotypes, sympatric differentiation is not favored (Buzato et al. 1994), even if some phenotypes are selected by one pollinator or the other.

For all phenotypes, UV reflectance was low, which corroborates the pattern mentioned by Domingos‐Melo et al. (2021) of UV‐absorbing white flowers pollinated by bats. Some phenotypes, however, presented low green reflection, rather resembling ornithophilous flowers (Coimbra et al. 2020) and likely visually inconspicuous to bats. Although color signals seem to present only a secondary role in this system, there is also the possibility of moderate, opposing selection on color, e.g., toward white by bats and red by hummingbirds, such that intermediate, whitish pink morphs gain combined fitness benefits. An analogous pattern, with opposing preferences maintaining an intermediate morph, has been reported for a color‐polymorphic orchid pollinated by bees selecting for white and flies selecting for black flowers, generating overdominance of the red morph, which is visited by both (Kellenberger et al. 2019). In our studied population, the dominant category of color phenotypes is UV‐White, indicating stronger selection by bats on green reflection, followed by UV‐Pink phenotypes, which presumably are selected by both, and some UV‐Black phenotypes, less abundant but probably maintained by bird selection, which present no innate color preferences but are conditioned to darker, redder flowers offering more rewards (Tavares et al. 2016; Coimbra et al. 2020). Together, these results indicate mixed selection since UV‐absorbing white flowers, optimal for detection at dusk, predominate in the population (sensu Domingos‐Melo et al. 2021), but still other darker, redder phenotypes persist, possibly linked to bird selection and maintained by the free flow of pollen between the different color phenotypes (Buzato et al. 1994).

Other flower signals such as flower shape, scent, and acoustic cues (Gonzalez‐Terrazas et al. 2016, Simon et al. 2011) may undergo stronger selection. In the case of bats, wider, bell‐shaped and acoustically conspicuous corollas (sensu Gonzalez‐Terrazas et al. 2016) may be more attractive, due to a higher reflective area, whereas for hummingbirds, deeper, tubular flowers may be an indicator of high rewards (Tavares et al. 2016). Although we cannot exclude the possibility of differential selection favoring specific floral phenotypes more suited to either bats or hummingbirds, especially the diurnal and nocturnal variation in corolla diameter (i.e., narrower during the day and wider at night) especially suggests that *C. sellowiana* is adjusted to both pollinator groups equally well. This functional flexibility may reduce any selective pressure toward floral specialization.

In *C. sellowiana*, nectar volumes of 27 ± 8.5 μL at 21% ± 8% concentrations in exposed flowers in the morning are well above that found for *Siphocampylus sulfureus* E. Wimm. (6.0 ± 7.4 μL, Sazima et al. 1994), a sympatric species also pollinated by bats and hummingbirds, with more diluted nectar (11.5% ± 3.9%). Compared to other hummingbird‐pollinated species of the Atlantic Forest and considering nectar measurements from excluded flowers, *C. sellowiana* presents more abundant but less concentrated nectar (114.0 μL at 15% vs. 34.3 ± 41.0 μL at 24% ± 12%, *n* = 150 spp., Tavares et al. 2016), resembling a bat‐pollinated species in that sense, such as *Hymenaea cangaceira* (196.63 ± 156.44 μL at 17.4% ± 3%, Domingos‐Melo, Diniz, et al. 2020) or other bat‐pollinated assemblages (two studied locations; 150.8 and 167.0 μL, at 15.0% and 18.1%, respectively, Sazima et al. 1999). These data suggest *Callianthe sellowiana* presents chiropterophilous nectar traits. Although we did not conduct chemical analyses, nectar odor is frequently reported as a characteristic trait of chiropterophilous flowers and may play an important functional role in attracting bats (Raguso 2004; Machado et al. 1998; Sazima et al. 1999; Domingos‐Melo, Milet‐Pinheiro, et al. 2020). Future studies addressing the chemical composition of nectar and its potential olfactory signaling in *C. sellowiana* would provide valuable insights into the role of scent in this bimodal pollination system.

The foraging behavior of distinct pollinator functional groups influences pollen deposition patterns, which can impact reproductive success (Muchhala 2003; Cárdenas‐Calle et al. 2020). Compared to hummingbirds, bats generally transport larger loads of more genetically diverse pollen (i.e., non‐related conspecific pollen) which can enhance reproductive success in self‐incompatible species (Muchhala and Thomson 2010). In *C. sellowiana*, pollen tube germination confirmed self‐incompatibility. At the stigma, no differences were detected among treatments, suggesting that both self‐ and cross‐pollen grains are able to germinate. However, at the middle and base of the style, open‐pollinated flowers showed higher pollen tube counts than all experimental treatments, indicating that natural pollen deposition by pollinators ensures greater tube development. Importantly, no statistical differences were observed between self‐pollination and the mixed or cross‐pollination treatments. Despite the fact that the species is self‐incompatible (unpublished data), it remains unclear at which stage incompatibility is fully expressed. One possibility is that the proportion of cross‐pollen grains in the experimental mixtures was insufficient to overcome the effect of self‐pollen, preventing detectable differences. Understanding whether the barrier occurs at late pollen tube development or at fertilization requires further investigation. The occasional shifts in the predominant pollinator group may confer reproductive advantages, as maintaining both pollinator groups could enhance reproductive success (Cárdenas‐Calle et al. 2020). While hummingbirds are frequent visitors, their territorial behavior (see Buzato et al. 1994) often results in high self‐pollen deposition, whereas bats may provide larger loads of genetically diverse pollen (Muchhala and Thomson 2010).

## 5 Conclusions

Based on the set of observed floral adaptations, we propose that *C. sellowiana* represents a case of a specialized bimodal system within the specialization‐generalization continuum. The species relies on pollination by both bats and hummingbirds to achieve higher seed set capacity. Its floral morphology facilitates effective contact with both functional groups by exhibiting a wider floral diameter at night and a narrower one during the day, three‐day anthesis, and abundant nectar available during the day and night (*sensu* Muchhala 2007), possibly also alternately enhancing visual and acoustic signals for each pollinator group. The species presents great color variation, but with equally conspicuous phenotypes, indicating low selective pressures on this trait or advantages to phenotypes that represent shared signals to both pollinators. Furthermore, the absence of statistical differences in fruit and seed formation between diurnal and nocturnal exclusion treatments indicates that both pollinator groups are equally effective. Additionally, the significantly higher seed and fruit production observed in the open‐pollination treatment, which included both nocturnal and diurnal pollinators, highlights the reproductive advantage conferred by maintaining both pollination strategies. These findings reinforce the role of functional and temporal complementarity in enhancing reproductive success in bimodal pollination systems and explaining the positive effects of biodiversity in pollination ecology. We highlight the importance that future studies on *C. sellowiana* measure the quality of pollen received by different pollinator groups due to the role of pollen quality for plant reproductive success and assess the role of scent in this pollination system.

## Author Contributions

**Isis Paglia:** conceptualization (equal), data curation (equal), formal analysis (equal), investigation (lead), methodology (equal), visualization (equal), writing – original draft (lead), writing – review and editing (lead). **Gabriel Coimbra:** data curation (equal), formal analysis (equal), investigation (supporting), methodology (supporting), visualization (equal), writing – original draft (supporting), writing – review and editing (supporting). **Leandro Freitas:** conceptualization (equal), funding acquisition (lead), methodology (equal), supervision (lead), writing – review and editing (lead).

## Conflicts of Interest

The authors declare no conflicts of interest.

## Supporting information


**Appendix S1:** ece372391‐sup‐0001‐AppendixS1.zip.

## Data Availability

All the required data is uploaded as Appendix S1.
