# Supplementary material for: Mixed Bat‐Hummingbird Pollination Assures Reproductive Success in a Highly Variable Upper Montane Species
Source: Ecol Evol. 2025 Oct 23;15(10):e72391. doi: 10.1002/ece3.72391 (PMC12549183; doi:10.1002/ece3.72391)
Supplement: Supplementary file 1 — Appendix S1: ece372391‐sup‐0001‐AppendixS1.zip. [file ECE3-15-e72391-s001.docx]

**Supplementary materials**

**Mixed bat-hummingbird pollination assures reproductive success in a highly variable upper montane species**

Isis Paglia^1,*^, Gabriel Coimbra^1^, Leandro Freitas^1^

^1^Jardim Botânico do Rio de Janeiro. Rua Pacheco Leão, 915. Rio de Janeiro, Rio de Janeiro, RJ, Brasil. CEP: 22460-030.

**Table S1 -** Corolla opening across anthesis stages. N = number of flowers measured. Opening (mm) reports the mean ± SD of individual measurements. SE (mm) is the standard error of the mean (SD/√N), reflecting precision of the mean estimate. 95% CI (mm) is the confidence interval for the mean, computed as mean ± 1.96 × SE; summarizing the uncertainty around the stage mean.

| **Stage** | **N** | **Opening (mm)** | **SE (mm)** | **95% CI (mm)** |
| --- | --- | --- | --- | --- |
| Night 1 | 8 | 43.29 ± 10.07 | 3.56 | 36.31–50.27 |
| Day 1 | 9 | 36.65 ± 5.02 | 1.67 | 33.37–39.94 |
| Night 2 | 5 | 37.57 ± 2.61 | 1.17 | 35.28–39.86 |
| Day 2 | 5 | 33.02 ± 2.59 | 1.16 | 30.74–35.29 |

**Table S2** - Per-phenotype spectral and visual metrics, including color category, N: number of reproductive individuals counted with similar phenotype, mean reflectance in the UV (300-400) and green (500-600 nm) wavebands, CCB (JND) (chromatic contrast against the leaf background under a hummingbird visual model), ACB (JND) (achromatic contrast against the leaf background/luminance contrast)

| **Phenotype** | **Category** | **N ind** | **UV** | **Green** | **CCB (JND)** | **ACB (JND)** |
| --- | --- | --- | --- | --- | --- | --- |
| Red | UV-Black | 3 | 0.02 | 0.24 | 5.25 | 0.32 |
| Salmon | UV-Pink | 2 | 0.02 | 0.32 | 1.53 | 0.17 |
| Yellow | UV-White | 4 | 0.02 | 0.58 | 1.34 | 0.10 |
| Grey | UV-White | 2 | 0.02 | 0.72 | 1.27 | 0.22 |
| White | UV-White | 2 | 0.01 | 0.87 | 1.25 | 0.32 |
| Whitish Pink | UV-White | 2 | 0.00 | 0.54 | 1.28 | 0.05 |
| Pink | UV-Black | 3 | 0.05 | 0.23 | 1.81 | 0.32 |
| Light Pink | UV-White | 3 | 0.06 | 0.77 | 1.38 | 0.27 |
| Medium Pink | UV-Pink | 3 | 0.05 | 0.40 | 1.46 | 0.07 |
| Dark Pink | UV-Pink | 3 | 0.11 | 0.10 | 2.01 | 0.41 |
| Purple | UV-Black | 2 | 0.03 | 0.04 | 1.18 | 0.60 |
| Blue | UV-Pink | 2 | 0.02 | 0.26 | 1.59 | 0.25 |

**Table S3** - One-way ANOVA models testing for differences in visual metrics among color categories (UV-White, UV-Pink and UV-Black).

| **Response** | **F** | **p (overall)** | **R²** | **Adj. R²** | **AIC** |
| --- | --- | --- | --- | --- | --- |
| UV | 1.25 | 0.332 | 0.217 | 0.043 | -46.57 |
| Green | 20.00 | <0.001 | 0.816 | 0.776 | -10.71 |
| CCB (JND) | 1.84 | 0.213 | 0.290 | 0.133 | 39.60 |
| ACB (JND) | 2.71 | 0.120 | 0.376 | 0.238 | -9.38 |

**Table S4 -** Pairwise category comparisons with Tukey-adjusted p-values; groups are full category names.

| **Response** | **Group 1** | **Group 2** | **Difference** | **p (adj)** |
| --- | --- | --- | --- | --- |
| UV | UV-Pink | UV-Black | 0.02 | 0.618 |
| UV | UV-White | UV-Black | -0.01 | 0.904 |
| UV | UV-White | UV-Pink | -0.03 | 0.309 |
| Green | UV-Pink | UV-Black | 0.09 | 0.614 |
| Green | UV-White | UV-Black | 0.52 | <0.001 |
| Green | UV-White | UV-Pink | 0.43 | 0.002 |
| CCB (JND) | UV-Pink | UV-Black | -1.10 | 0.392 |
| CCB (JND) | UV-White | UV-Black | -1.44 | 0.195 |
| CCB (JND) | UV-White | UV-Pink | -0.35 | 0.875 |
| ACB (JND) | UV-Pink | UV-Black | 0.19 | 0.219 |
| ACB (JND) | UV-White | UV-Black | 0.22 | 0.114 |
| ACB (JND) | UV-White | UV-Pink | 0.04 | 0.920 |

**Table S5 -** Fruit and seed production per treatment in *Callianthe sellowiana* at Itatiaia National Park. Number of flowers, number of fruits, fruit set (percentage of flowers that developed into fruits), and mean ± standard deviation of the number of seeds per fruit in each pollination treatment.

| **Treatment** | **Flowers (n)** | **Fruits (n)** | **Fruit set (%)** | **Seeds per fruit (mean ± SD)** |
| --- | --- | --- | --- | --- |
| C | 30 | 18 | 60.0 | 25.3 ± 5.2 |
| DE | 28 | 16 | 57.1 | 23.8 ± 4.7 |
| NE | 32 | 17 | 53.1 | 24.1 ± 6.1 |
